# Phytoextraction of Cr(VI)-Contaminated Soil by *Phyllostachys pubescens*: A Case Study

**DOI:** 10.3390/toxics9110312

**Published:** 2021-11-18

**Authors:** Ezio Ranieri, Gianfranco D’Onghia, Francesca Ranieri, Andrea Petrella, Vincenzo Spagnolo, Ada Cristina Ranieri

**Affiliations:** 1 Dipartimento di Biologia, Università di Bari, 70125 Bari, Italy; gianfranco.donghia@uniba.it; 2 Dipartimento di Giurisprudenza, Università di Bari, 70121 Bari, Italy; fra.ranieri97@gmail.com; 3 Dipartimento Dicatech, Politecnico di Bari, 70125 Bari, Italy; andrea.petrella@poliba.it; 4 Dipartimento Interateneo di Fisica, Politecnico di Bari, 70125 Bari, Italy; vincenzoluigi.spagnolo@poliba.it (V.S.); cristinaranieri4@gmail.com (A.C.R.); 5 Università Telematica Internazionale Uninettuno, 00186 Roma, Italy

**Keywords:** bamboo growth, tolerance, Cr removal, metal translocation, phytoextraction, *Moso bamboo*

## Abstract

This work presents the results of experimental tests to evaluate the effects of prolonged contamination by Cr on *Moso Bamboo* (*MB*) (*Phyllostachys pubescens*) and the adaptability of the *MB* to the Mediterranean climate. A preliminary test on the *MB* was developed in the laboratory, simulating irrigation under Mediterranean conditions (600 mm per year) and tropical conditions (1800 mm per year), to evaluate the rate of growth and the *MB*’s capability for Cr phytoextraction from contaminated soil. The tolerance of *MB* to Cr was also performed showing a good response of the plant to 100 mg Cr/L solution, utilized for irrigation of the pots. The results show that the rate of *MB*’s removal of Cr from soil ranged from 49.2% to 61.7% as a function of the soil degree of contamination, which varied from approx. 100 mg/kg to 300 mg/kg. The distribution of Cr in the various sections of the bamboo revealed that the greater percentage was present in rhizomes: 42%, equal to 114 mg Cr for 600 mm per year, and 50%, equal to 412 mg Cr for 1800 mm per year. A noteworthy diffusion of the metal towards the outermost parts of the plant was shown. The values of Cr retained in the stems and leaves of *MB* tissues were quite high and varied from 1100 mg/kg to 1700 mg/kg dry weight.

## 1. Introduction

Phytoremediation is one of the best alternatives to conventional physicochemical remediation technologies, which produce secondary pollution, are highly expensive, and can deteriorate soil fertility [1,2,3,4]. For phytoextraction, model plants should possess a wide root apparatus with a high yield of biomass in the presence of an elevated heavy metal mass [5].

Chromium is required as a nutrient for human metabolism with consumption of 100 µg daily, but excessive exposure to Cr(VI) has toxic effects on human health, particularly on the respiratory system [6,7,8,9,10]. Chromium represents a wide concern in soil contamination, including in Italy [11].

**Figure 1:**
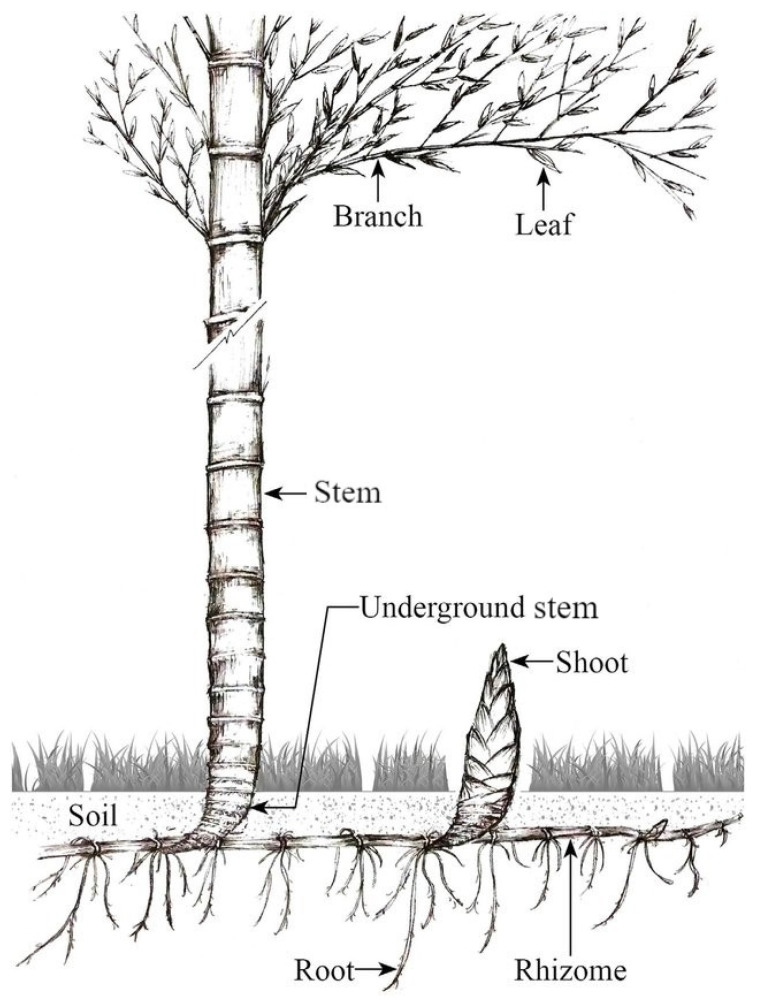
Roots, rhizome, stem, and leaves in *MB* (*Phyllostachys pubescens*) [49].

In phytoremediation, plants used in phytoextraction, such as *Jerusalem artichoke* [12] and *Moso Bamboo* (*MB*)—*Phyllostachys pubescens* (Figure 1)—should exhibit good transfer of the metal from the roots to the aboveground parts (measured via the translocation factor), must be resistant and accumulate high levels of heavy metal (measured via the bioconcentration factor), and should have a rapid growth rate [13].

Plant growth is not inhibited for a soil concentration up to 100 mg Cr/kg dry weight [14,15,16,17]. However, bamboo plants show some negative effects in respect to growth and in plant organs for metal-contaminated soils with more than 300 mg/kg dry weight [5].

Bamboo species had a survival rate of 100% for all the species grown for a soil concentration of around 100 mg Cr/kg [18]. Therefore, it is supposed that MB should tolerate a higher metal stress, and there is potential to use MB as a phytoremediation material for Cr-contaminated soil up to 200–300 mg Cr/kg.

In experiments with 1000 mg/kg Cr(VI), stem growth was found to be drastically reduced for 94% of examined plant species [19], confirming that Cr excess can cause many plant metabolic changes and reduce their growth [20]. However, in some plants, Cr at a low level (0.05–1.0 mg L^−1^) promotes growth and yield, especially in crops, but it has not been proven essential to plants [21,22]. Plants use different mechanisms to control the toxic effects of Cr, accumulating it in their tissues through uptake via the roots and subsequent translocation [23].

Chromium phytoextraction depends on the specific hyperaccumulator–contaminant interaction [24,25,26,27,28,29].

Plants that accumulate more than 500 mg Cr/kg in dry leaf tissue can be considered hyperaccumulators [30]. They can stabilize Cr in the soil through their roots and aboveground tissues by one or several mechanisms, converting it to Cr(III) [31].

MB is easily adaptable to a typical Mediterranean climate [32] and it has shown a great ability to accumulate metals with high biomass production; this occurs via the plant’s ability to uptake metals that are essential for its growth. This implies, in the case of positive results at the experimental level, easy applicability of the intervention at full scale [26,33,34,35,36,37,38,39,40,41,42,43].

The growth rates of the culms of bamboo species have earned this group of plants the status of being the fastest-growing grasses on Earth [44]. MB rapidly reaches its maximum size with an average height of 15 m. Bamboo shoots rise above the ground within 35–40 days, reaching 15–20 m in height and 10–12 cm in diameter [45].

MB is characterized by high biomass productivity, ease in cultivation, extensive competitive ability, a short cutting time (4–5 years), and multiple uses such as for furniture, building materials, and decoration. Furthermore, it is known as the most promising species for carbon sequestration [46,47], and it has a high mean aboveground carbon sequestration value (8 ± 2.15 MgC ha^−1^ year^−1^) [48].

On the basis of our previous work [11], wherein an experimental study was carried out in the laboratory to evaluate *MB*’s tolerance and capacity for Cr phytoextraction and was developed in three stages.

The aim of this work was to confirm *MB*’s growth rate, resistance, and phytoextraction capacity in Cr-contaminated soil over a prolonged contamination time.

A results comparison across the two experiments allowed us to evaluate the differences in the plant response as a consequence of Cr irrigation time during the entire process.

Specifically, in this study, the use of *MB* for phytoremediation was investigated for the following purposes:

To recognize its ability to develop in different environments;

To evaluate *MB*’s tolerance in Cr-contaminated soils;

To quantify its long-term Cr phytoextraction capacity;

To evaluate the migration capacity of Cr within the plant;

To assess the difference in the Cr content in plant sections as a function of different phytoextraction timings.

## 2. Materials and Methods

The three different phases of the experiment all had the same duration of 84 days. The first phase objective was to compare the development of *MB* in an environment differing from that where it originated, characterized by a tropical or subtropical climate, and its development in a typical Mediterranean climate. Week by week, growth was monitored through the measurement of the growth rates of the main morphological parts of the plant, such as stems and leaves.

During the second phase, a tolerance evaluation was conducted through the measurement of *MB*’s growth in soil contaminated by a Cr(VI) solution. Changes in the morphological structures were measured weekly, allowing the growth rates to be calculated under stress.

During the last 84 days—the third phase—the amount of Cr(VI) that had moved from the soil to the various parts of the plant was evaluated, the main plant areas affected by increased accumulation were identified, and the results were translated into a mass balance.

The specimens were taken from *MB* plants and soil purchased from Tiberio Tricarico Ltd. Garden Centre (Terlizzi, Bari, Italy). Different types of tests—specifically, adaptability, contamination, and soil Cr(VI) sorption tests—were carried out for the roots, rhizomes, stems, and leaves of the plants.

The soil medium was characterized by a mixture of peat (blond/brown) and vegetable conditioner and showed a density of approximately 0.25 kg/L, C(org) ≅ 20% dry weight (dw) N ≅ 1% dw, pH ≅ 6.9. The four pots employed for the experimental campaign (diameter = 250 mm, height = 200 mm, volume = 10 L) were filled with approximately 4 kg of soil.

To contaminate the soil in the four pots, K_2_Cr_2_O_7_ was used in the appropriate quantity to form an aqueous solution of 100 mg Cr/L [18].

The irrigation throughout the first two steps was carried out with tap water showing the following chemical composition: HCO_3_^−^ = 270 mg/L; NO_3_^−^ = 8 mg/L; Ca^2+^ = 30.9 mg/L; K^+^ = 27.7 mg/L; F^−^ = 1 mg/L; Mg^2+^ = 9.5 mg/L. The rainfall regime and the diameter of the soil bed were the parameters used to evaluate the irrigation volume. Specifically, the experimental flows (Q) for the growth and contamination tests were obtained in accordance with the rainfall regimes associated with climates in the Apulia region (1.64 mm/d) and tropical areas (4.93 mm/d) using the same type of soil but irrigated with different amounts of water; accordingly, the values were Q_1_ = 0.0805 L/d for the first case and 0.242 L/d for the second case.

### 2.1. Analysis of the Bamboo Growth

An estimation of the bamboo growth was obtained after measuring the plant height every week. The measurements started when the plants reached the following heights:

57.2 cm and 54.9 cm for Pots 1 and 2, respectively, for *MB* growth with 1.64 mm/d tap water.

97.2 cm and 96.5 cm for Pots 3 and 4, respectively, for *MB* growth with 4.93 mm/d tap water.

The elongation of a single element, cluster, or whole stem from each underground rhizome system was recorded by a camera, which allowed us to evaluate the actual growth of the bamboo plants.

### 2.2. Sample Preparation and Characterization

The selected samples were submitted to chemical digestion after the following preliminary steps. In the first step, a thermal treatment at 75 °C was carried out for moisture removal, followed by mixing and sieving (2 mm mesh) processes. Afterwards, the different parts of bamboo as roots, rhizomes, stems, and leaves were obtained after separation and were washed with tap and demi-water to remove the excess soil grains; then, they were dried at 75 °C. Finally, a milling process was carried out in order to obtain particles of approximately 0.2 mm, which were chemically digested. Specifically, a mixture of HNO_3_ and HCl (7:1) was used for the digestion of 0.5 g of sample in a PFA/TFM oven (4.93 W), and the solution was then diluted to 50 mL.

A 20 mL mixture of HNO_3_ and HCl (1:4) was added to 1.5 g of dried soil specimens in a digestion tube. The operations were carried out at 160 °C in a fume chamber by a digestion block and stopped when the final volume of solution was approximately 4 mL. The addition of 20 mL of HNO_3_ and HCl (1:4) solution continued until a final volume of 5 mL was reached by thermal treatment. Afterwards, the solution was filtered (10 µm membrane Isopore Merck filters) and the liquid was diluted to 25 mL. Finally, the solutions of the digested samples together with the references were characterized by ICP-OES in order to obtain the total Cr concentration (milligrams per kilogram of dry weight (DW)). Specifically, the Cr values were obtained in the soil before *MB* transplanting, whereas the Cr determinations of rhizosphere soils, roots, rhizomes, stems, and leaves were carried out six months after *MB* transplanting. All chemicals were purchased from Aldrich, were of suprapur grade, and were used as received without further purification or distillation.

### 2.3. Statistical Analysis

The samples were analyzed in triplicate, and the data obtained are reported as the mean ± standard deviation. In order to evaluate statistically significant differences among values, all data regarding wet and dry weight measurements and the Cr contents of plant tissues (roots, rhizomes, stem, and leaves) were analyzed via one-way ANOVA and post hoc Tukey’s test (*p* < 0.05). A two-way repeated-measures ANOVA was used to analyze the relationship between plant height and stem diameter growth, treatment, and time.

## 3. Results and Discussion

### 3.1. Growth Rate Test on MB

Adaptation tests were carried out prior to the growth test to verify the ability of the plants to adapt to a climate different from that of their natural habitat. The 84-day growth test was carried out in a laboratory-controlled environment, in which the following parameters were constantly monitored: soil pH (6.9), light exposure (14 h light and 10 h dark), temperature (20 °C), and optimal irrigation volume (1.64 mm/d for pots 1,2 and 4.93 mm/d for pots 3,4).

**Figure 2:**
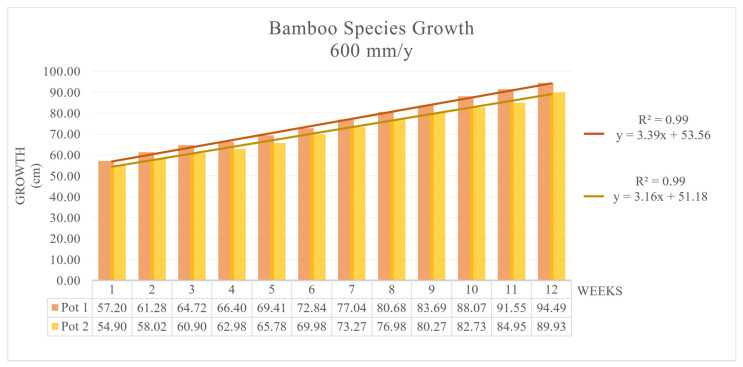
*MB* growth with 1.64 mm/d tap water, Pots 1 and 2.

**Figure 3:**
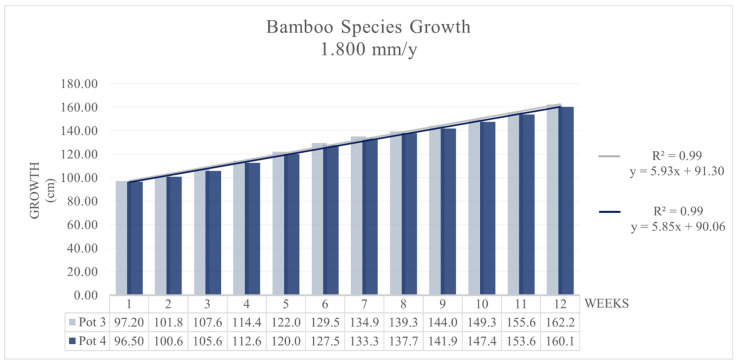
*MB* growth with 4.93 mm/d tap water, Pots 3 and 4.

The growth rate, intended to represent the growth rates of the different morphological parts and the overall plant, was defined through length variation measurements carried out weekly. The results are shown in Figure 2 for Pots 1 and 2 and in Figure 3 for Pots 3 and 4. In both cases, the bamboo plants showed good adaptation capacity. The growth rate g_r_ was very similar for Pots 1 and 2 (1.64 mm/d) and averaged 3.29 cm/week; for Pots 3 and 4 (4.93 mm/d), g_r_ was 5.85 cm/week on average. The interpolation curve was linear in the four cases: h = 3.39 (weeks) + 53.56 with R^2^ > 0.99 for Pot 1; h = 3.16 (weeks) + 51.18 with R^2^ = 0.9964 for Pot 2; h = 5.933 (weeks) + 91.302 with R^2^ = 0.996 for Pot 3; and h = 5.854 (weeks) + 90.057 with R^2^ > 0.99 for Pot 4. For both climate cases, the growth rate g_r_ was lower than that in the previous experiment [11], where the growth rate g_r_ was 4.56 cm/week for Pots 1 and 2 and 8.45 cm/week for Pots 3 and 4. The total elongation, similar in both pots for each irrigation rate, also seems to be lower than that observed in natural conditions for *MB* [50,51].

### 3.2. Contamination and Tolerance Test

The treatment with the contaminant lasted about three months according to the concentration of the contaminant solution. Otherwise, low metal exposure (<100 mg kg^−1^) does not inhibit plant growth in pot experiments [52].

At the end of our lab tests, the data obtained were the weekly growth rate for each plant and the morphological development of the plants after the contamination, revealed by analysis of the Cr adsorbed in samples of the different structures of the plant. At a concentration of 100 mg Cr/L in irrigation water, in both Pots 1 and 2, the bamboo plants still maintained their vegetative functions.

**Figure 4:**
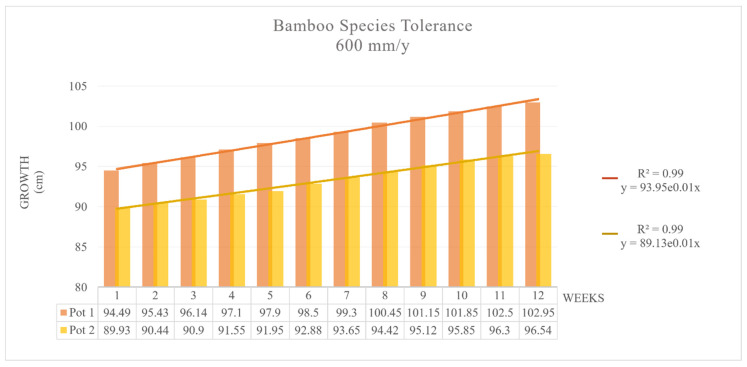
MB tolerance: Growth with 1.64 mm/d contaminated water, Pots 1 and 2.

In the first pot, the growth interpolation curve was h = 93.94 × 10^0.008 (weeks)^ with R^2^ > 0.99, and the second pot had the equation h = 89.132 × 10^0.007 (weeks)^ with R^2^ > 0.99 (Figure 4).

Similar to in Pots 1 and 2, *MB* in Pots 3 and 4, where the soil was contaminated with an aqueous solution of 100 mg Cr/L, does not show symptoms of contamination stress.

**Figure 5:**
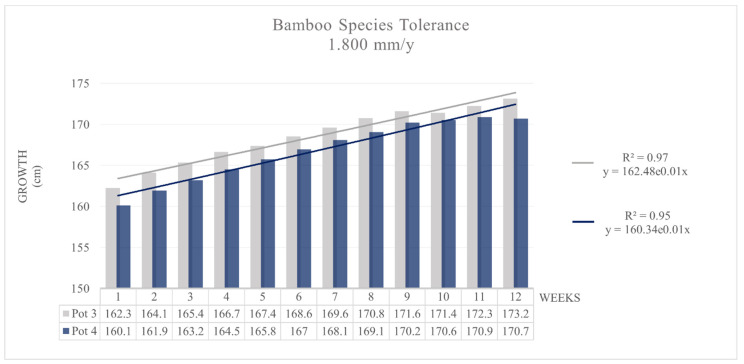
MB tolerance: Growth with 4.93 mm/d contaminated water, Pots 3 and 4.

Bamboo tolerance in Pots 3 and 4 was higher than that under the 1.64 mm/d conditions, and a higher g_r_ was also revealed. The interpolation curve for Pot 3 was h = 162.48 × 10^0.0056 (weeks)^ with R^2^ = 0.9672; the same curve for Pot 4 was h = 160.34 × 10^0.0061 (weeks)^ with R^2^ = 0.9514 (Figure 5). In both cases, there was an increase in plant tolerance compared to that in the previous experiment [11], probably due to the higher volume of biomass developed [53,54,55,56].

The administration of Cr induced significant changes in tissue morphological parameters after the fifth or sixth week. These consequences affected the roots and rhizomes more than the stem and leaves, with a greater volume reduction and a more pronounced decrease in the number of new shoots.

### 3.3. Cr Phytoextraction from the Soil

Previous contamination tests [11] also aimed to quantify how much Cr the *MB* plant is able to retain, considering that approx. 40% of the flow containing Cr leached away from the bottom of the pot. Chromium belongs to the second class of hazard according to the Russian general toxicological standard for soil [11].

**Figure 6:**
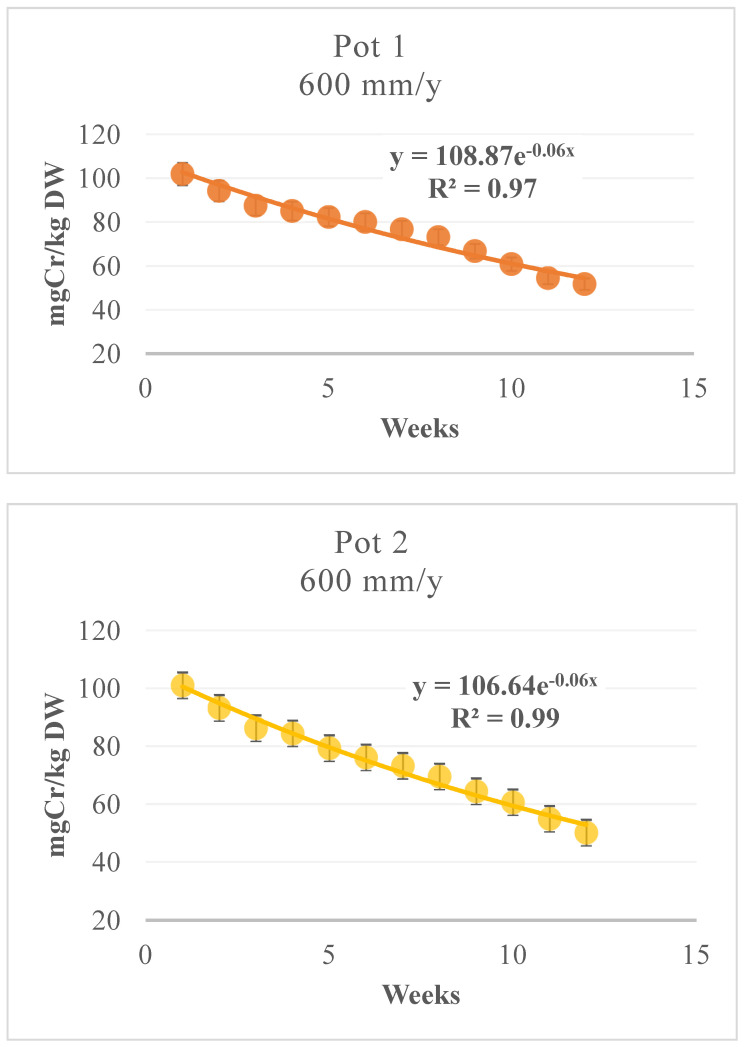
*MB* phytoextraction: Growth with 1.64 mm/d contaminated water, Pot 1–Pot 2.

**Figure 7:**
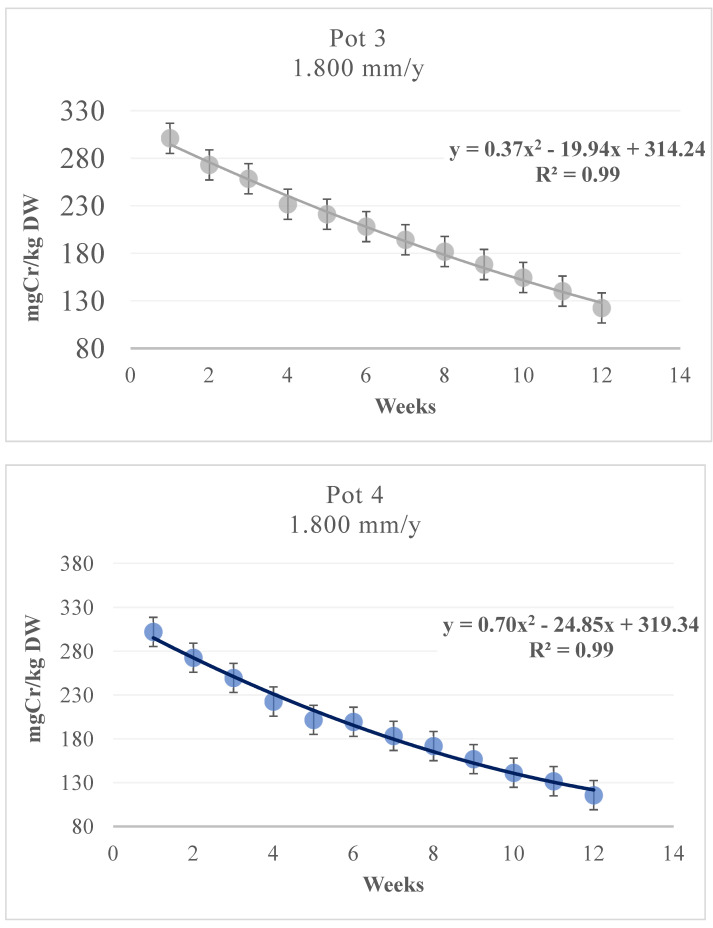
*MB* phytoextraction: Growth with 4.93 mm/d contaminated water, Pot 3–Pot 4.

Figure 6 shows the phytoextraction capacity of bamboo and the soil Cr content after three months for the 1.64 mm/d pots; Figure 7 shows the same for the 4.93 mm/d pots. The residual concentration of Cr in the soils after the experimentation ranged from 50.1 to 51.8 mg/kg for Pots 2 and 1, respectively (Figure 6), and from 115.7 to 122.5 mg/kg for Pots 4 and 3, respectively (Figure 7). The soil analysis results indicated that the Cr levels were reduced considerably by the plants growing on the contaminated soils, with a removal rate of 50%—similar to those in other experiments [57] and higher than that in the previous study [11], in which the Cr removal percentages from soil ranging from 43.3 to 44.7% starting from a contamination of approx. 105 mg/kg dw for 600 mm/year and ranging from 44.3 to 47.5% starting from a contamination of approx. 295 mg/kg dw for 1800 mm/year, in 12 weeks.

The percentage of Cr removal from soil ranged from 49.2% to 50.4% (Pots 1 and 2) when starting from a contamination of approximately 102 mg/kg, and it ranged from 59.3% to 61.7% (Pots 3 and 4) when starting from a contamination of approximately 302 mg/kg. The interpolating curve for Pot 1 was [Cr] = 108.87 × 10^−0.058 (weeks)^ with R^2^ = 0.962, and that for Pot 2 was [Cr] = 106.64 × 10^−0.058 (weeks)^ with R^2^ = 0.982 (Figure 6). For Pots 3 and 4, the corresponding curves were [Cr] = 0.3682(week)^2^ −19.943(week) + 314 with R^2^ = 0.99 and [Cr] = 0.6989(week)^2^ −24.852(week) + 319 with R^2^ = 0.99 (Figure 7), respectively, showing a higher tendency to continue phytoextraction over time. The soil characteristics significantly influence the Cr absorption capacity of the plant. In fact, in soil rich in humid acids, the metal is predisposed to creating bonds with acids in a preferential way, thus reducing the phytoextraction capacity of the plant. This has been confirmed by other studies [58], and the phenomenon involves a reduction in bioavailability of up to 57% [23].

The results confirm that a greater presence of biomass is associated with greater efficacy in phytoextraction and a greater amount of extracted Cr [43,53].

### 3.4. Cr Distribution in Tissues

**Figure 8:**
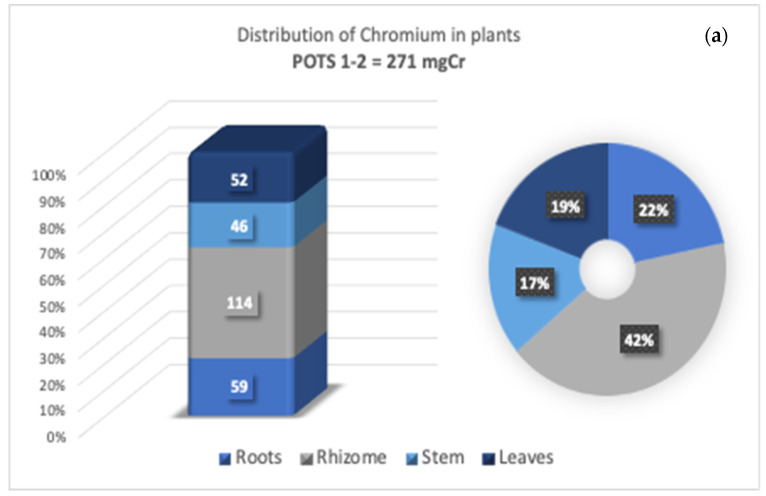

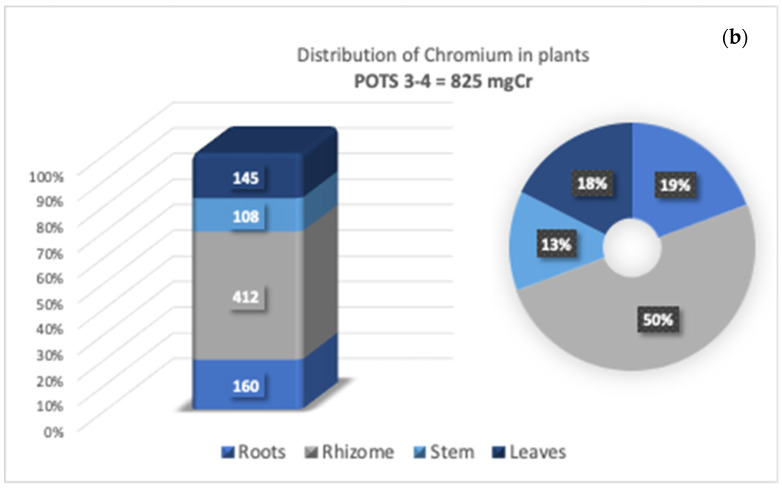
Extraction of Cr expressed in milligrams (**left**) and as a percentage (**right**) accumulated in the different parts of MB, under 600 mm/y (**a**) 1800 mm/y (**b**) irrigation.

After the contamination test, samples of roots and leaves were taken from all plants to determine the distribution of metal in the plant tissues. The Cr concentrations in the aerial parts of the plants were found to be low; this could be due to the fact that the poisoning period was insufficient to exceed the tolerance limit of the roots and rhizomes and therefore did not allow the time necessary for the translocation of the metal to the outer parts [59]. The higher concentration of metal in the roots and rhizomes confirmed that *MB* concentrates Cr principally in the rhizome–root system by limiting transport to the aerial parts—parts included in the animal and human food chain [60,61,62,63]. In Figure 8a,b, the distribution of the Cr in the bamboo tissues is shown for 600 mm/y and 1800 mm/y, respectively. As shown in Figure 8a, Cr was mainly accumulated in the root–rhizome apparatus, at a level of approximately 173 mg Cr, representing 64% of the total Cr retained. Comparable percentages (Figure 8b) of Cr were revealed in Pots 3 and 4, irrigated with 4.93 mm/d, with a slightly lower translocation rate and where the Cr content in leaves was approximately 18%. These results show some differences from the previous work [11], where an average of 69% of Cr was detected in the rhizome and 18% in the roots, while only 6 % and 7% were found in the stem and leaves, respectively. In this case, the percentages stored by the outermost parts of the plant grew significantly to 19%; conversely, the presence of Cr in the rhizomes was reduced by up to 46%, on average. The variations indicate greater diffusion of Cr inside the plant over time, perhaps due to the plant’s adaptation to metal, which implies a sort of predisposition of the plant to store Cr during growth in all sections of the plant. Other studies have reported that bamboo accumulates three-quarters of its entire biomass in only 40 days, and this happens in the initial growth stage [64].

**Figure 9:**
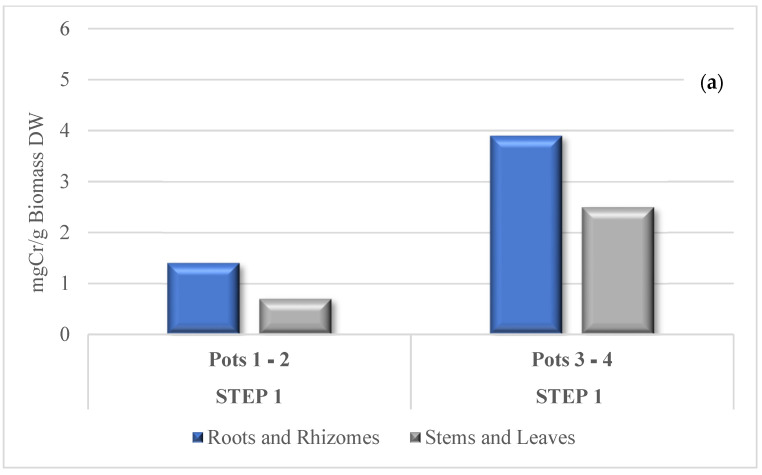

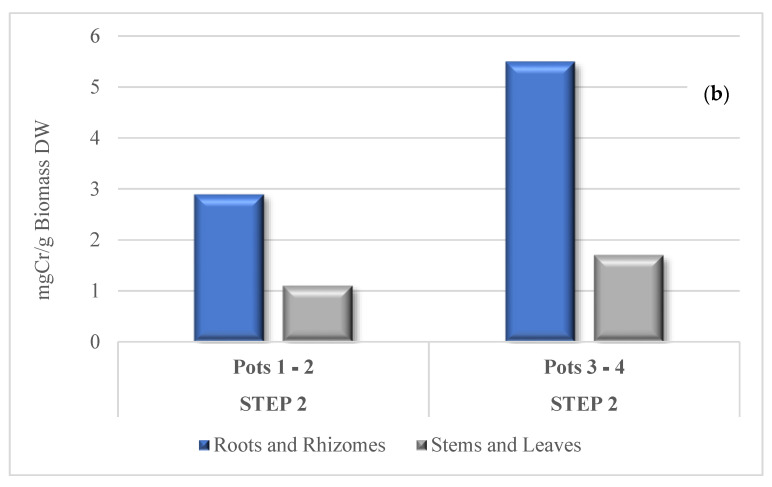
Absorbed milligrams of Cr per gram of biomass in a previous experiment [11] (**a**) and in the present experiment (**b**).

Figure 9 reports a comparison between the results of this investigation and the previous experiment [11] in terms of milligrams of Cr adsorbed per gram of biomass dw in the different sections of the plant. The values of Cr retained in the *MB* tissues were quite high and varied for stems and leaves from 1100 mg/kg dw to 1700 mg/kg dw and from 2900 mg/kg dw to 5500 mg/kg dw for roots and rhizomes, respectively for pots 1–2 and for pots 3–4 without showing toxic effects that could alter physiological and metabolic pathways [65]. These values are comparable with those for other hyperaccumulator species such as *Cannabis sativa* and *Allium griffithianum*, which can accumulate higher concentrations of Cr ranging from 568.33 to 1233.3 mg/kg [66], and *Diectomis fastigiata*, which can accumulate Cr in roots at about 2371 mg/kg, while *Vernonia cinerea* can accumulate 5500 mg/kg dry weight Cr in shoots [67].

The values in milligrams of Cr per gram of biomass shown are slightly lower than the values observed in the previous experiment [11], particularly for Pots 3 and 4, due to the greater development of biomass, and they should reflect higher tolerance to Cr. Moreover, it should be noted that there is a tendency of the plant to store other Cr during subsequent growth, confirming the possibility for *MB* to act as a hyperaccumulator of the metal.

## 4. Conclusions

*Moso Bamboo* showed good growth in lab conditions under 1.64 mm/d irrigation conditions, with an average value of 3.29 cm/week, and under 4.93 mm/d irrigation conditions, with an average value of 5.85 cm/week.

The results regarding the tolerance of the plant to Cr showed a good response of the plant to 100 mg Cr/L solution, utilized for irrigation of the pots. Plant growth during exposure to Cr was reduced to about 1 cm/week, as average.

The Cr removal percentages were also noteworthy, showing that the rate of *MB*’s removal of Cr from soil ranged from 49.2% to 61.7% as a function of the soil degree of contamination.

The values of Cr retained in the *MB* tissues were quite high and varied for stems and leaves from 1100 mg/kg dw to 1700 mg/kg dw and from 2900 mg/kg dw to 5500 mg/kg dw for roots and rhizomes, respectively.

*Moso Bamboo* showed good adaptability to the Mediterranean climate and high performance in the removal of Cr from soil with potential application on large-scale remediation sites.
